# PD-1, PD-L1, and PD-L2 Expression as Predictive Markers in Rare Feline Mammary Tumors

**DOI:** 10.3390/vetsci12080731

**Published:** 2025-08-03

**Authors:** Maria Franco, Fernanda Seixas, Maria dos Anjos Pires, Anabela Alves, Andreia Santos, Carla Marrinhas, Hugo Vilhena, Joana Santos, Pedro Faísca, Patrícia Dias-Pereira, Adelina Gama, Jorge Correia, Fernando Ferreira

**Affiliations:** 1 CIISA—Center of Interdisciplinary Research in Animal Health, Faculty of Veterinary Medicine, University of Lisbon, 1300-477 Lisboa, Portugal; 2 Associate Laboratory for Animal and Veterinary Sciences (AL4AnimalS), 1300-477 Lisboa, Portugal; 3 Veterinary Sciences Department, University of Trás-os-Montes and Alto Douro (UTAD), 5000-801 Vila Real, Portugal; 4 Animal and Veterinary Research Centre (CECAV), UTAD, 5000-801 Vila Real, Portugal; 5 ICBAS-UP, School of Medicine and Biomedical Sciences, University of Porto, 4050-313 Porto, Portugal; 6 Animal Science and Study Centre/Food and Agrarian Sciences and Technologies Institute (CECA/ICETA), 4050-478 Porto, Portugal; 7 Centre for Investigation Vasco da Gama (CIVG), Department of Veterinary Sciences, Vasco da Gama University School, 3020-210 Coimbra, Portugal; 8 Onevetgroup Hospital Veterinário do Baixo Vouga (HVBV), 3750-742 Águeda, Portugal; 9 DNAtech Veterinary Laboratory, 1649-038 Lisboa, Portugal; 10 Faculty of Veterinary Medicine, Universidade Lusófona de Humanidades e Tecnologias, 1749-024 Lisboa, Portugal; 11 Laboratório Associado para a Química Verde (LAQV), Rede de Química e Tecnologia (REQUIMTE), University of Porto, 4050-453 Porto, Portugal

**Keywords:** PD-1/PD-L1/PD-L2 immunoregulatory axis, biomarkers, tumor expression, immunotherapy

## Abstract

Feline mammary carcinoma (FMC) exhibits aggressive behavior, with limited treatment options. Given the relevance of the PD-1/PD-L1/PD-L2 axis in human breast cancer immunotherapy, this study assessed PD-1 and its ligands in rare FMC histotypes (n = 48) using immunohistochemistry on tumor cells (TCs), intratumoral lymphocytes (iTILs), and stromal tumor-infiltrating lymphocytes (sTILs). PD-1 was expressed in 13% of TCs, 85% of iTILs, and 94% of sTILs, while PD-L1 was observed in 46% of TCs, 96% of iTILs, and 100% of sTILs. PD-L2 was expressed in 79% of TCs and 100% of both iTILs and sTILs, with PD-L1/PD-L2 co-expression in 42% of TCs. Higher PD-1 IHC scores in TCs were associated with a less aggressive biological behavior; PD-L1 in iTILs was linked to skin ulceration, whereas PD-L2 in TCs was associated with its absence. Our findings highlight the relevance of the PD-1/PD-L1/PD-L2 immune checkpoint in rare FMC subtypes and support further investigation into checkpoint-blockade therapies.

## Simple Summary

Mammary carcinoma is a common tumor in cat, showing high mortality, sharing several clinicopathological features with human breast cancer, and with few therapeutical options. In recent years, the development of drugs targeting the PD-1/PD-L1/PD-L2 immunomodulatory axis has shown very promising results in the treatment of breast cancer. Thus, in this study, the expression of PD-1, PD-L1 and PD-L2 was analyzed in tumor cells and in tumor-infiltrating lymphocytes of rare feline mammary carcinomas, as scarce data are available regarding special histotypes. In addition, statistical associations between the expression levels and clinicopathological characteristics were tested. The obtained results uncover the importance of the PD-1/PD-L1/PD-L2 axis in these tumors and support further research on molecular targeted therapies.

## 1. Introduction

Feline mammary carcinoma (FMC) is the third most common type of tumor in this species, showing a high mortality and metastasizing rate [1,2]. Apart from common histological types, a group of malignancies with heterogeneous features is also identified, comprising a panoply of rare and uncommon tumors, also frequently associated with poor prognosis, that include simple micropapillary and anaplastic carcinomas, and special types of carcinoma, such as adenosquamous, mucinous, lipid-rich, inflammatory carcinoma, and carcinosarcoma. Despite the scarcity of information on this group of tumors, a recent multicentric study of 1778 feline mammary tumors identified fifty-four rare feline mammary tumors (3.04%), including twelve adenosquamous (0.68%), eleven mucinous (0.62%), ten carcinosarcomas (0.56%), nine anaplastic (0.51%), six inflammatory carcinomas (0.38%) and six micropapillary tumors (0.33%), showing that the incidence of rare feline mammary tumors is not negligible [3]. The diagnosis is usually made at an advanced stage of the disease [4] and the currently available therapeutic options for FMC are very limited, with the most widely accepted being the unilateral or bilateral radical mastectomy with removal of the respective lymph nodes, or their combination with adjuvant chemotherapies [1,5]. However, although radical surgical procedures are associated with longer disease free-survival (DFS), there is no significant effect on overall survival (OS) rates [6]. Regarding the drugs used in the context of chemotherapy, such as doxorubicin or cyclophosphamide, these tend to have limited efficacy, especially after metastasis has occurred, and may cause adverse side effects [1,6]. Thus, the discovery of novel diagnostic markers and therapeutic targets is needed to improve the clinical management of feline mammary carcinoma [1,7].

In recent years, exceptionally promising results have been observed in clinical trials carried out in human oncology, using immune checkpoint inhibitors (ICIs) in various types of malignant neoplasms [8,9], including human breast cancer [10]. Immune checkpoints correspond to a set of immunoregulatory pathways that help maintain self-tolerance, prevent autoimmunity, and mitigate possible collateral damage to tissues [11] through a biological orchestra guided by the reception of co-stimulatory or co-inhibitory signals that regulate the functions of immune cells. From this perspective, the PD-1/PD-L1/PD-L2 pathway plays a key role in sending co-inhibitory signals to activate T cell functions under physiological conditions [12]. However, tumor cells have acquired the ability to hijack this pathway, using it as a “molecular shield” to escape immune system (IS) attack, hindering the ability of immune cells to recognize and control tumor progression [11].

The programmed cell death protein-1 (PD-1) is frequently expressed by infiltrating CD8+ and CD4+ T lymphocytes, natural killer T cells, B cells, activated monocytes and dendritic cells [13], acting as receptor and activated through interaction with its specific ligands: programmed death ligand-1 (PD-L1) and programmed death ligand-2 (PD-L2) [14]. PD-L1 expression occurs at low levels in B and T cells, dendritic cells, mast cells, macrophages, epithelial cells, endothelial cells and in certain types of tumor cells (TCs) [11], and is not expressed in healthy breast tissue [15]. In parallel, the PD-L2 expression is limited to dendritic cells, B cells, macrophages [16] and sometimes expressed by TCs [17], sharing 40% of its sequence identity with PD-L1 [11].

Notably, the recognition that the PD-1/PD-L1/PD-L2 immunomodulatory axis was involved in tumor progression provided scientific rationale for the development of drugs, namely monoclonal antibodies, which specifically target the PD-1/PD-L1/PD-L2 axis and are capable of significantly regenerating the antitumor functions of T cells [18,19]. Accordingly, a monoclonal antibody which blocks PD-L1 binding to the PD-1 receptor on T cells was approved by the Food and Drug Administration to treat several tumor types and PD-L1 positive unresectable locally advanced and metastatic triple negative (TN) breast cancer [19], with many studies showing that PD-1 and PD-L1 are highly expressed in HER2-positive and TN cancer subtypes [20,21,22,23,24]. Furthermore, the VENTANA’s PD-L1 assay was also approved as a complementary diagnostic test to assess patients’ PD-L1 expression prior to treatment [20]. Indeed, a recent comprehensive meta-analysis showed a strong association between PD-L1 expression in TCs and unfavorable prognostic outcomes [25]. It has been pointed out, however, that PD-L1 expression in tumor-infiltrating lymphocytes (TILs) could potentially be a favorable prognostic biomarker [20,25]. As far as PD-L2 is concerned, studies carried out in human oncology have shown that overexpression of this ligand is associated with poorer prognosis in different tumors [26]. However, to date, no clinical trials have demonstrated the efficacy of immunotherapy targeting the PD-L2 ligand, thus further research is still needed.

In veterinary medicine, immunotherapy targeting the PD-1/PD-L1 pathway has already demonstrated antitumor efficacy in canine malignant oral melanoma and undifferentiated sarcoma [27,28]. However, to date, no commercial antibody targeting the PD-L1/PD-L2 ligands has been approved for dog and/or cat. Nevertheless, given the extensive homology between the immune systems and oncogenic mechanisms among mammals, Maekawa et al. hypothesize that ICI immunotherapy represents a promising strategy for the treatment of neoplasms in cats [29]. In addition, they have also pointed out that the high sequence and structure similarity in PD-1, PD-L1 and PD-L2 domains in different mammalian species strongly suggests a conserved function of PD-1/PD-L1/PD-L2 axis in T cell-mediated immune response suppression [29].

Considering the above evidence and the fact that rare feline mammary tumors have not been investigated either due to their relative rarity, or probably to limited inter-observer agreement in their diagnosis, in this study we aimed to (i) evaluate the expression of PD-1, PD-L1 and PD-L2 in the tumor cells and TILs (intratumoral and stromal) of rare feline mammary carcinomas and (ii) explore possible associations between IHC scores and various clinicopathological characteristics.

## 2. Material and Methods

### 2.1. Immunohistochemical Staining and Analysis

For the analysis of PD-1/PD-L1/PD-L2 expression, three sections with 3 μm thickness (Microtome Leica RM2135, Newcastle, UK) of formalin-fixed paraffin-embedded tissues were prepared, mounted on adhesive slides, and placed at 64 °C overnight. Then, deparaffinization, rehydration and epitope retrieval were performed using a PT-Link module (Dako, Agilent, Santa Clara, CA, USA), by immersing glass slides in Antigen Target Retrieval Solution at pH 9 (Dako, K8000) for anti-PD-L1 antibody and pH 6 (Dako, K8000) for anti-PD-1 and anti-PD-L2 antibodies, over the course of 40 min at 96 °C. Thereafter, the slides were cooled for 20 min at room temperature (RT) and rinsed twice for 5 min in distilled water. The endogenous peroxidase activity was blocked by an incubation period of 15 min with Peroxidase Block Novocastra Solution (Novocastra, Leica Biosystems, Newcastle, UK), and, after two washing steps with PBS, the nonspecific binding of immunoglobulins was prevented by incubating the tissue slides with the protein block Novocastra solution (Leica Biosystems) for 15 min at RT. Then, before two PBS washes (2 × 5 min), tissue slides were incubated with an anti-PD-1 monoclonal antibody (dilution 1:25, clone J116, eBioscience, San Diego, CA, USA), or with an anti-PD-L1 monoclonal antibody (dilution 1:100, clone CAL 10, Abcam, Waltham, MA, USA), or with an anti-PD-L2 polyclonal antibody (dilution 1:400, #PA5-20344, Invitrogen, Carlsbad, CA, USA) for 60 min at RT, followed by further washes with PBS (2 × 5 min). Post primary Novocastra solution (Leica Biosystems) was then used to saturate any binding sites still available in the target tissue that had not previously been blocked by the primary antibody, for a period of 30 min at RT. After further washing with PBS (2 × 5 min), incubation with Novolink Polymer (Leica Biosystems) was carried out for 30 min. Subsequently, and after a supplementary PBS wash, the staining was achieved by a 5 min incubation with the DAB Chromogen Solution (Dako, K8000) diluted in Novolink DAB substrate buffer (Dako, K8000). Ultimately, FMC tissue sections were counterstained with Harris hematoxylin for 1 min, dehydrated and mounted (VectaShield, Vector Laboratories, Newark, CA, USA).

TILs were accessed according to the recommendations proposed by the International Immuno-Oncology Biomarkers Working Group [30]. Briefly, TILs should be reported separately for the stromal compartment (% stromal TILs) and the tumor cell compartment (% intratumoral TILs), although in breast carcinoma the decision might be made to assess only stromal TILs. TILs should only be evaluated within the limits of the invasive tumor, and as a continuous variable, and tumor areas with technical artifacts, necrosis or hyalinization should be excluded. In addition, all mononucleated cells should be counted, but neutrophilic polymorphonuclear leukocytes should be excluded. One section (3 μm, magnification 200–400×) per tumor is considered sufficient; however, it is recommended to evaluate additional sections for each case whenever possible, due to tumor heterogeneity [30].

Thus, to assess PD-1, PD-L1 and PD-L2 expression in TILs and tumor mammary cells, the tissue samples were evaluated in 7–9 individual fields at 400× magnification: 3–4 fields for observing intratumoral TILs (iTILs), defined as positively labeled mononucleated cells in contact with tumor cells or in the intima of tumor cell nests [30]; 3–4 fields for the analysis of stromal TILs (sTILs), referring to positively stained mononucleated cells in the interstitial stroma surrounding the TCs [30]; and 1 field for individual TC assessment. The variability in the number of fields examined was due to the intention to evaluate several cells equal to or greater than 100 for each of the subpopulations evaluated, ensuring a more accurate representation of these proteins in tumor tissue, considering intratumoral heterogeneity.

The percentage of labeling was calculated by dividing the number of positively labeled cells by the total number of cells (marked and unmarked) incorporated into the fields observed and multiplied by 100. For positive cells (PP), the scores of percentages were recorded as follows: 0 (<1%), 1 (1–5%), 2 (6–30%), and 3 (>30%). In parallel, for intensity, the scores (IS) were as follows: 0 (negative), 1+ (weak), 2+ (moderate), 3+ (strong) and 4+ (very strong). Finally, the percentage of positive cells (PP) and intensity scores (IS) were multiplied to calculate a final IHC score, ranging 0–12. Given that, to date, there are no guidelines for cut-off values, the final IHC score of the expression of each of the molecules (PD-1, PD-L1 and PD-L2) equal to or greater than 1 (PP × IS ≥ 1) was considered positive when evaluating TCs and TILs (intratumoral and stromal). This is similar to previous studies which considered the expression of at least 1% of cells with any intensity to be positive [31,32,33].

Feline lymph node tissues were used as positive controls, whereas sections of healthy mammary tissues were used as negative controls. All slides were independently subjected to blind scoring by two independent pathologists, who were responsible for the histological diagnosis.

### 2.2. Statistical Analysis

The statistical analysis was carried out in IBM SPSS Statistic version 29.0.0.0 for MacOS. A *p* value of <0.05 was considered statistically significant for a 95% confidence interval. The histograms were plotted in SPSS and the remaining graphs in GraphPad Prism version 10.2.3 for MacOS. The tables were made in Microsoft Excel for MacOS. Firstly, the distribution of the dependent variables to be compared was checked, i.e., the immunohistochemistry scores of PD-1, PD-L1 and PD-L2 expressed by the TCs, iTILs and sTILs, to decide on the next tests to be applied. To this end, normality tests were carried out, specifically the Shapiro–Wilk test. The Spearman’s coefficient test, used for non-parametric data, with n > 30 was applied to explore possible correlations between the expression of the 3 proteins in TCs, iTILs and sTILs. To compare the expression of PD-1, PD-L1 and PD-L2 molecules in tumor cells and TILs with different locations (intratumoral and stromal) among patients with different histological types of tumors, breeds, ages and grades of malignancy, the Kruskal–Wallis test and Dunn’s multiple comparisons post-test were applied. The Mann–Whitney test was used to compare the expression levels of PD-1, PD-L1 and PD-L2 in TILs and TCs with various clinicopathological characteristics, such as the size of the mass, skin ulceration, tumor necrosis, lesion distribution, vascular permeation, and regional lymph node invasion. The results were presented as median values.

## 3. Results

### 3.1. Animals

The animal population enrolled in this study included cats with rare mammary tumors such as invasive micropapillary carcinoma, anaplastic carcinoma, lipid-rich carcinoma, mucinous carcinoma, adenosquamous carcinoma, inflammatory carcinoma and carcinosarcoma, diagnosed during a 12-year period (2011–2022), at the Pathology Laboratory of the Faculty of Veterinary Medicine/ULisbon, and accordingly to the classification proposed by Zappulli et al. [34]. Mammary tumor tissue samples that were not adequately preserved, or that did not follow strict processing protocols, such as a fixation time in 10% formalin for 48 h or less, were excluded from this study. Thus, 47 queens were included, and 48 samples of rare mammary carcinomas were collected, as one of the patients had two histologically distinct types of tumors. The following clinicopathological characteristics were recorded: age at diagnosis, breed, tumor size, presence of skin ulceration, histopathological classification, tumor histological grade, presence of tumor necrosis, lesion distribution (unifocal or multifocal), vascular permeation, lymph node status (Table 1), and additionally, disease free-survival (DFS) and overall survival (OS).

Briefly, the mean age at diagnosis was 12.2 years (range 4–20 years), in which most patients (42/47, 89%) were aged 8 years or more at the time of diagnosis. According to Table 1, the predominant breed was the European shorthair (33/47, 70%), while it was found that 81% (39/48) of the tumors were 2 cm or larger. In addition, 79% (38/48) of the mammary tumors showed no skin ulceration.

As for the histopathological classification of the 48 samples, 36 (75%) were mucinous carcinomas, followed by 8 (17%) adenosquamous carcinomas and 4 (8%) carcinosarcomas (Table 1). The average DFS was 10.0 months (n = 15; 95% CI: 3.4–16.4 months) and the average OS was 12.7 months (n = 15, 95% CI: 6.0–19.4 months).

### 3.2. PD-1, PD-L1 and PD-L2 Are Expressed by Tumor Cells and TILs

**Figure 1:**
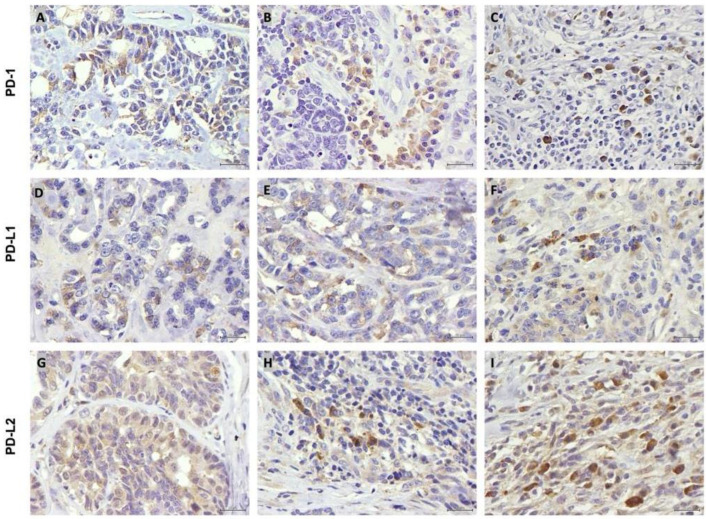
Representative PD-1, PD-L1 and PD-L2 immunolabeling of tumor cells (TCs) and of intratumoral (iTIL) and stromal (sTIL) tumor-infiltrating lymphocytes (TILs) in rare histotypes of feline mammary carcinomas (×400, bar = 20 μm). (**A**) Positive PD-1 immunostaining of TCs showing a moderate intensity score (2+); (**B**) a weak intensity score of iTILs (1+) and (**C**) a strong intensity score of sTILs (3+). (**D**) PD-L1 immunolabeling of TCs showing a score intensity of 2+ or (**E**) 3+, with iTILs exhibiting an intensity score of 2+ (**F**). (**G**) PD-L2 labelling of TCs (score 1+), (**H**) iTILs (score 2+) and (**I**) sTILs (score 2+).

The IHC analysis revealed that rare histotypes of FMC showed a positive PD-1 expression in the cytoplasm and cell membrane of TCs, intratumoral and stromal TILs (Figure 1A–C). In parallel, PD-L1 expression was mainly detected in the cytoplasm and nuclear membrane of TCs (Figure 1D,E), whereas the intratumoral and stromal TILs exhibited a positive cytoplasmic and a cell membrane staining pattern (Figure 1E,F). Regarding the PD-L2 expression, TCs displayed a cytoplasmic and nuclear distribution pattern (Figure 1G), while both subpopulations of TILs showed cell membrane/cytoplasmic immunoreactivity and/or nuclear staining (Figure 1H,I), showing low expression in spindle cells (Figure 1H), in endothelium (Figure 1I) and macrophages.

### 3.3. PD-1 Is More Highly Expressed in TILs than in Tumor Cells

**Figure 2:**
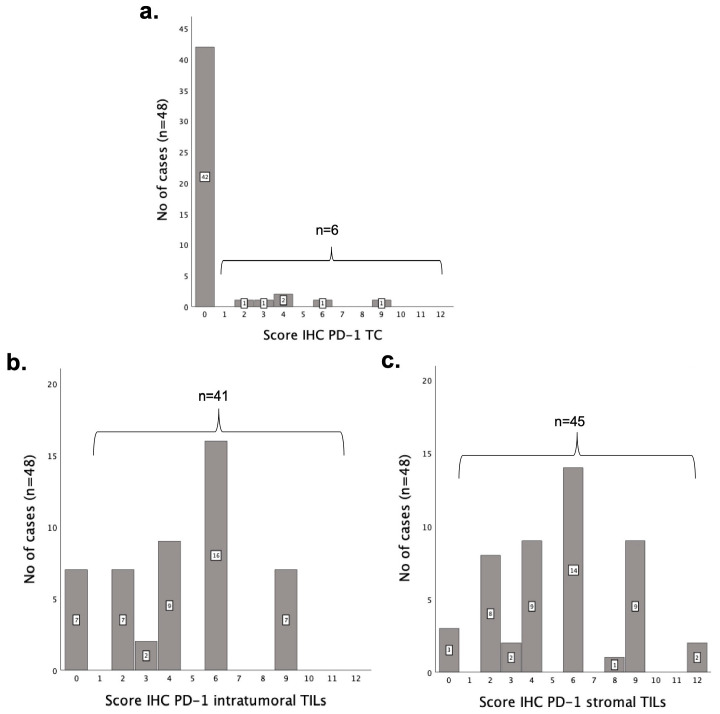
Histograms of final IHC scores for PD-1 in (**a**) tumor cells (TCs), (**b**) intratumoral TILs and (**c**) stromal TILs. The “n” located above the brackets corresponds to the number of cases in which the immunolabeling was positive (≥1).

Given that final IHC scores ≥ 1 were considered positive, 13% of the samples (6/48) were positive for PD-1 expression in TCs (Figure 2a). Regarding the intratumoral and the stromal TILs, PD-1 expression was detected in 85% (41/48) and 94% (45/48) of tumors, respectively (Figure 2b,c), with 6% (3/48) of the samples showing no PD-1 immunoreactivity for the three cell subpopulations analyzed.

### 3.4. PD-L1 Is Expressed at Lower Levels than PD-L2 Both in Tumoral Cells and TILs

**Figure 3:**
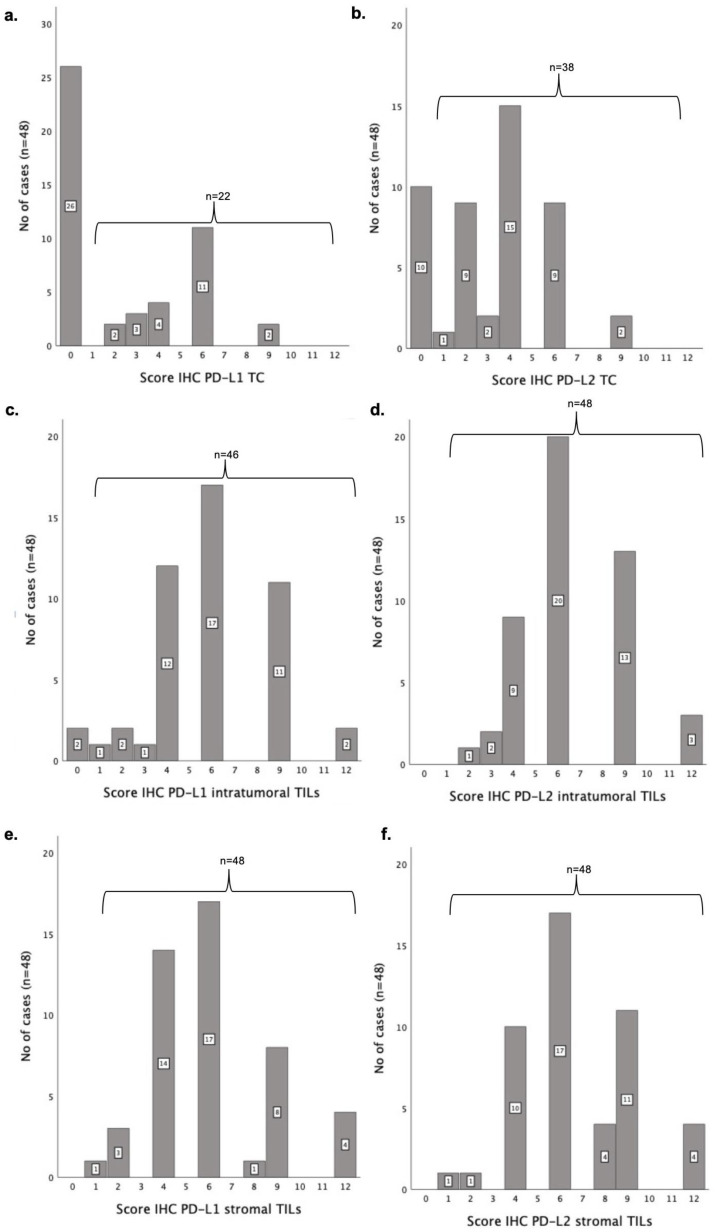
Histograms of PD-L1 and PD-L2 IHC scores in tumor cells (**a**,**b**), in intratumoral tumor-infiltrating lymphocytes (**c**,**d**) and in stromal TILs (**e**,**f**). The “n” on the brackets corresponds to the number of tumor samples with a positive IHC score (≥1).

The expression of the PD-L1 ligand was highly prevalent in the analyzed tumor samples, with a positive IHC score in TCs identified in 46% (22/48) samples (Figure 3a), whereas 96% (46/48) and 100% (48/48) of the tumor samples showed positive scores in intratumoral and stromal TILs, respectively (Figure 3c,e). In addition, a high final IHC score (≥6) was found in TILs (63%; 30/48) and TCs (59%; 13/22), emphasizing that PD-L1 overexpression occurs in most of the tumor samples. Regarding the PD-L2 ligand, its expression in TCs was detected in 79% (38/48) of the tumor samples (Figure 3b), and in intratumoral (Figure 3d) and stromal (Figure 3f) TILs of all analyzed samples (100%; 48/48).

As with the PD-L1 ligand, PD-L2 expression in iTILs and sTILs was found to have a final IHC score ≥ 6 in most of the cases (75%; 36/48; Figure 3d,f). Notably, the PD-L2 expression was found in a greater number of samples than PD-L1 expression, with 42% (20/48) of tumors showing coexpression of both ligands on TCs.

### 3.5. PD-1, PD-L1 and PD-2 IHC Scores Are Correlated with Each Other and with Cell Type

**Figure 4:**
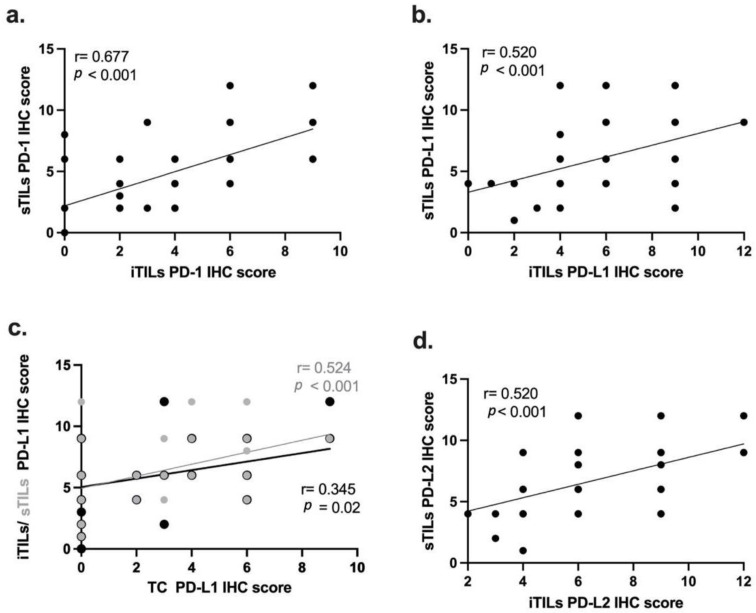
Spearman’s correlations of PD-1, PD-L1 and PD-L2 IHC scores in tumor cells (TCs), intratumoral tumor-infiltrating lymphocytes (iTILs) and stromal tumor-infiltrating lymphocytes (sTILs). Moderate and positive correlations were found in PD-1 IHC scores (r = 0.677; *p* < 0.001; (**a**)), PD-L1 IHC scores (r = 0.520; *p* < 0.001; (**b**)) and PD-L2 IHC scores (r = 0.520; *p* < 0.001; (**d**)) of iTILs and sTILs. In addition, a positive correlation between PD-L1 IHC scores of TCs and iTILs was detected (r = 0.345; *p* = 0.02; (**c**)) and of TCs and sTILs (r = 0.524; *p* < 0.001).

The results obtained show a positive, strong and significant correlation between PD-1 IHC scores found in iTILs and sTILs (r = 0.677; p ˂ 0.001; Figure 4a). Furthermore, positive, moderate and significant correlations were also found between PD-L1 IHC scores in iTILs and sTILs (r = 0.520; *p* ˂ 0.001; Figure 4b), between PD-L1 IHC scores in TCs and iTILs (r = 0.345; *p* = 0.02; Figure 4c) and between PD-L1 IHC scores in TCs and sTILs (r = 0.524; *p* ˂ 0.001; Figure 4c). Finally, a positive and moderate correlation was observed between PD-L2 IHC scores found in iTILs and sTILs (r = 0.520; *p* ˂ 0.001; Figure 4d).

**Figure 5:**
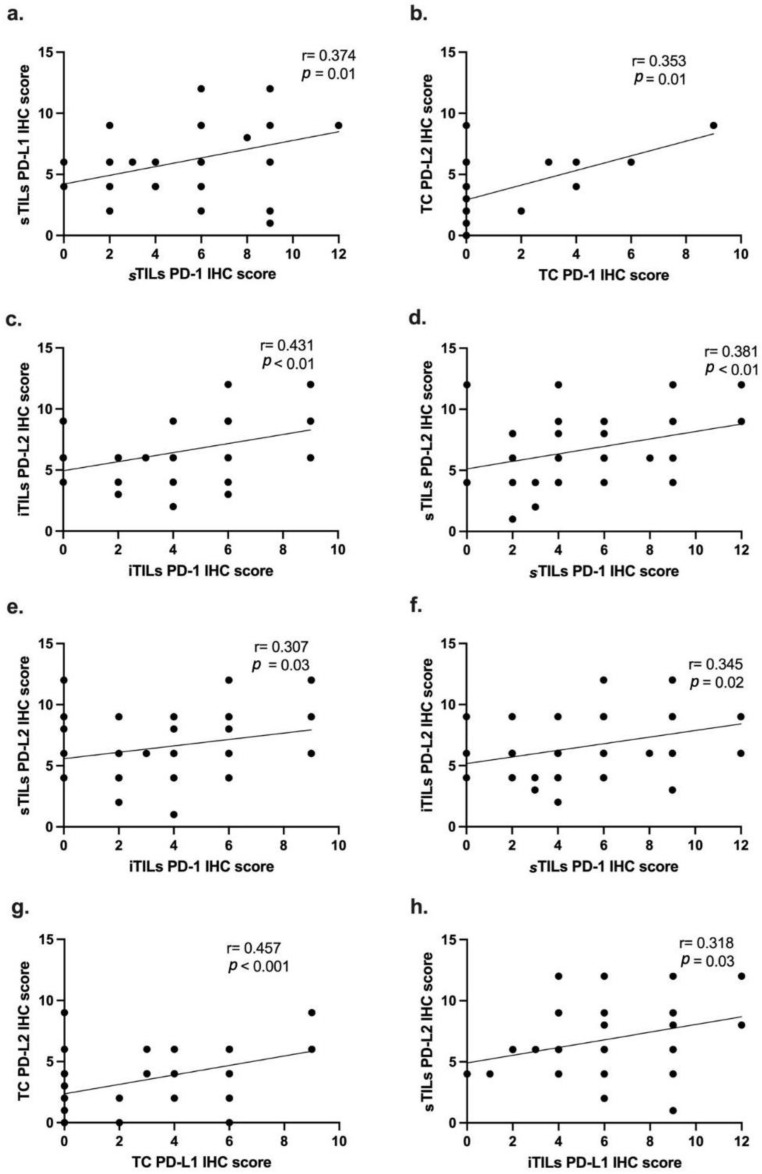
PD-1, PD-L1 and PD-L2 IHC scores are positively and significantly correlated in TCs, iTILs and sTILs. (**a**) The PD-1 IHC scores in sTILs showed a positive and moderate correlation with PD-L1 IHC scores in sTILs (r = 0.374; *p* = 0.01); (**b**) furthermore, PD-1 IHC scores in TCs are correlated with PD-L2 IHC scores in TCs (r = 0.353; *p* = 0.01) as with (**c**) PD-1 and PD-L2 IHC scores in iTILs (r = 0.431; *p* < 0.01) and (**e**) in sTILs (r = 0.307; *p* = 0.03). In addition, (**d**) PD-1 expression in sTILs showed a positive correlation with PD-L2 IHC scores in sTILs (r = 0.381; *p* < 0.01) and (**f**) in iTILs (r = 0.345; *p* = 0.02). Lastly, PD-L1 IHC scores in TCs (**g**) were positively correlated with PD-L2 IHC scores also in TCs (r = 0.457; *p* < 0.001), whereas PD-L1 expression levels in iTILs (**h**) were positively correlated with PD-L2 expression in sTILs (r = 0.318; *p* = 0.03).

Next, we investigated possible correlations between PD-1, PD-L1 and PD-L2 IHC scores in the different cell types. Statistical analysis revealed a positive and moderate correlation between PD-1 and PD-L1 IHC scores in sTILs (r = 0.374; *p* = 0.01; Figure 5a). Regarding the PD-1 and PD-L2 IHC scores, a positive and moderate correlation was found in TCs (r = 0.353; *p* = 0.01; Figure 5b). The Spearman’s coefficient test also revealed that PD-1 expression in iTILs is positively correlated with PD-L2 expression in iTILs (r = 0.431; *p* < 0.01; Figure 5c) and in sTILs (r = 0. 307; *p* = 0.03; Figure 5e). In addition, PD-1 IHC scores in sTILs were also found to be positively correlated with PD-L2 IHC scores in sTILs (r = 0.381; *p* < 0.01; Figure 5d) and iTILs (r = 0.345; *p* = 0.02; Figure 5f). Finally, a positive and moderate correlation was found between PD-L1 and PD-L2 expression levels in TCs (r = 0.457; *p* < 0.001; Figure 5g), as well as between PD-L1 expression in iTILs and PD-L2 expression in sTILs (r = 0.318; *p* = 0.03; Figure 5h).

### 3.6. Higher PD-1 IHC Scores in TCs Are Associated with Less Aggressive Clinicopathological Features

**Figure 6:**
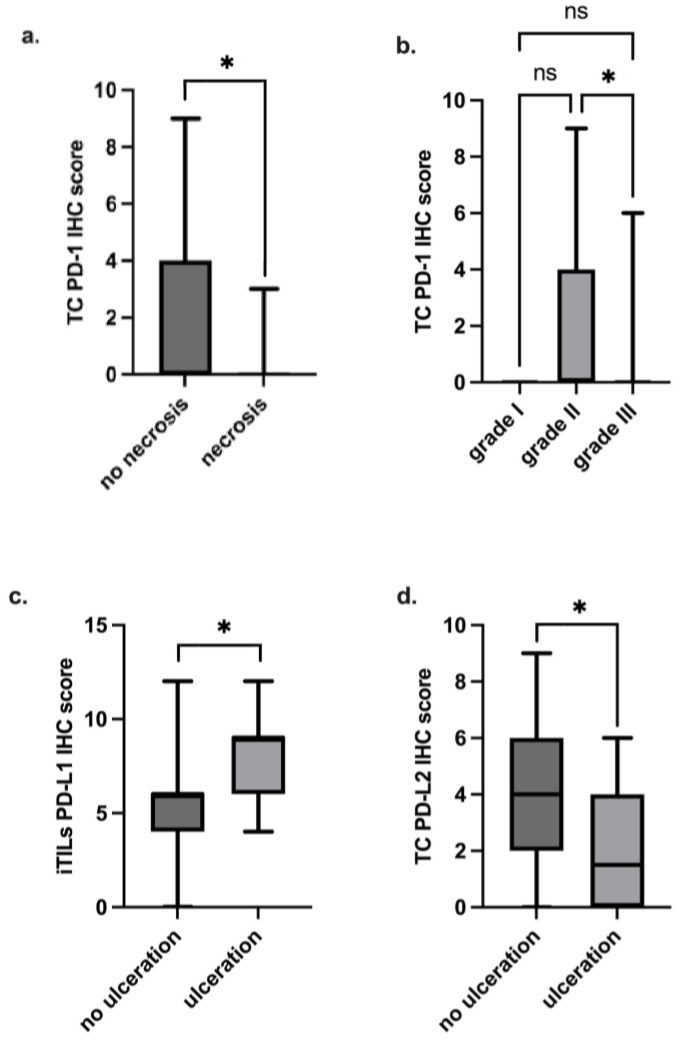
Box-plot analysis of PD-1, PD-L1 and PD-L2 IHC scores in TCs, iTILs and sTILs, and their association with some clinicopathological features. (**a**) Higher PD-1 IHC scores in TCs were found to be associated with tumors without necrosis and (**b**) with tumors with grade II in comparison with grade III tumors. (**c**) Higher PD-L1 IHC scores in iTILs were found to be associated with ulcerated tumors, while (**d**) higher PD-L2 IHC scores in TCs were found to be associated with tumors without skin ulceration. (* *p* < 0.05; ns—not significant).

Statistical analysis was then performed to investigate associations between the PD-1, PD-L1 or PD-L2 IHC scores in different cell types (TCs, iTILs and sTILs) and clinicopathological features presented by the animals enrolled in this study. Thus, it was found that PD-1 scores in TCs are associated with clinical characteristics related to a less aggressive disease course. Indeed, higher PD-1 IHC scores are associated with tumors without necrosis (Figure 6a and Table 2). Additionally, the Kruskal–Wallis (H) test reveals differences in PD-1 expression between histological grades II and III [H (2) = 8.788; *p* < 0.05], with grade III tumors showing lower IHC scores in TCs than grade II tumors (Figure 6b and Table 2).

### 3.7. PD-L1 and PD-L2 IHC Scores Are Significantly Associated with Some Clinicopathological Features

Regarding PD-L1, the Mann–Whitney U test showed that tumors with skin ulceration had higher IHC scores in intratumoral TILs in comparison with those without ulceration (*p* < 0.05; Figure 6c and Table 3). In parallel, tumors with no skin ulceration showed higher PD-L2 IHC scores in TCs when compared with those with ulceration (*p* < 0.05; Figure 6d and Table 3). No further significant associations were found (Table 2 and Table 3).

## 4. Discussion

Mammary tumors represent the third most common type of neoplasm in feline species, showing high mortality rates, partly due to the limited efficacy of currently available therapies. In humans, immunotherapy using monoclonal antibodies targeting the PD-1/PD-L1/PD-L2 axis has revolutionized the treatment of malignant neoplasms, including metastatic triple-negative breast cancer, with astonishing results. Interestingly, FMC shares several epidemiological, clinicopathological and histopathological characteristics, in addition to a molecular classification with HBC [7,35], raising the hypothesis that knowledge about new targeted therapies can be shared between the two species. However, very little is known about the role of the PD-1/PD-L1/PD-L2 axis in feline tumors and, to date, no therapeutic antibody to block this immunoregulatory axis has been developed for this species. Thus, exploring and evaluating the PD-1 expression and its ligands in feline mammary carcinoma samples is a crucial and important step.

Our results show that, in rare histotypes of feline mammary carcinoma, the PD-1 expression in TCs is detected in 13% (6/48) of analyzed samples. Although most researchers have focused on PD-1 evaluation on TILs, recent data have shown that PD-1 can also be expressed in TCs by a secondary mechanism, as identified in non-small cell lung carcinoma (NSCLC), colon carcinoma, melanoma, hepatocellular carcinoma, pancreatic adenocarcinoma, and triple-negative mammary carcinoma [36]. So far, the molecular mechanism that supports PD-1 expression in TCs is still unknown; however, it is speculated that changes in the number of gene copies, epigenetic modulation and/or disruption of tumor microenvironment may be involved [37]. Our study also revealed that higher PD-1 IHC scores in TCs were associated with a less aggressive biological behavior (e.g., absence of tumor necrosis, histological grade II instead of grade III).

Though these associations may seem counterintuitive, as PD-1 expression is often associated with immune exhaustion and consequent tumor progression [38], Du et al. have shown that, in lung carcinoma patients with high PD-1 IHC scores in TCs, the disease progressed rapidly after initiation of anti-PD-1 therapy [39], suggesting that PD-1 expression in TCs could potentially play a determining role in suppressing tumor progression when coupled with PD-L1 or PD-L2 ligands expressed on other TCs. Indeed, although, in colon carcinoma, the higher PD-1 scores in TCs were found to be associated with a lower pathological stage [40], in TN breast cancer, PD-1 expression in TCs promotes epithelial–mesenchymal transition (EMT), tumor growth and metastasis [41]. Indeed, several works on different human cancers correlated high PD-L1 expression with enhanced epithelial–mesenchymal transition, an initial phase of cancer spread, with cancer cells acquiring specific mesenchymal phenotypes, including morphological changes, expression of mesenchymal surface markers and related transcription factors, also suggesting a crosstalk between PD-L1 expression and chemoresistance [42,43]. In addition, researchers have suggested that this dual role of PD-1 can be explained by its interaction with SHP-1 and SHP-2 phosphatases, through the immunosuppressive functional motifs (ITIM and ITSM) localized at the small cytoplasmic domain of PD-1. Though it has already been reported that the ITIM and ITSM motifs remain conserved in the feline PD-1 receptor, signals for T cell suppression have not been investigated experimentally [27], indicating the need for more studies to fully elucidate the mechanisms of action of PD-1 in feline mammary tumors. Nevertheless, our statistical results between tumor malignancy grade and PD-1 score in TCs need to be validated by further studies due to the low number of collected tumors showing a positive PD-1 score.

Our results also show a high prevalence of rare histotypes of FMC with positive PD-1 IHC scores both in iTILs (85%, 41/48) and sTILs (94%, 45/48), as reported in human breast cancer [44], human gastric carcinoma [45] and Hodgkin’s lymphoma [46]. In the context of tumors, PD-1 expression is maintained at high levels, leading to a state of T cell exhaustion, with progressive loss of function and proliferative capacity [38], contributing to immunosuppressive tumor microenvironment (TME), and to promote epithelial–mesenchymal transition [47]. Taking our PD-1 results into account, cats with rare mammary carcinomas (e.g., mucinous carcinomas, adenosquamous carcinomas and carcinosarcomas) could be strong candidates for immunotherapy with ICI, namely antibodies directed against the PD-1/PD-L1/PD-L2 axis, which could reactivate the functions of T lymphocytes, restoring the anti-tumor activity [48].

Regarding PD-L1, several studies have reported its localization in cellular membrane [21,49,50], while others, in line with our findings, have reported its localization in both cytoplasm and nuclear membrane [7,17,51,52]. In addition, the PD-L1 expression levels vary considerably in HBC, ranging from 1.7% to 64% [28], partly due to the lack of a standard IHC technique, to the variety of available antibodies, and/or to the different cut-off values used. Recently, Li et al. have reported a PD-L1 immunoreactivity of 46.1% (231/501) in TCs of HBC [53] with similar results subsequently published by others. Nowadays, it is well accepted that, as part of an anti-tumor immune response, TILs, namely cytotoxic T cells (CD8+), release cytokines, including INF-γ, which in turn induces PD-L1 expression by TCs [54]. Additionally, CD8+ T cells have been shown to be key players in the tumor microenvironment in feline mammary carcinoma [55,56] and a recent study has shown that treatment with INF-γ induces PD-L1 expression by macrophages and mammary adenocarcinoma cell lines [27]. Moreover, PD-L1 expression exhibits a bidirectional relationship with the epithelial–mesenchymal transition, with factors of EMT promoting PD-L1 expression, while PD-L1 signaling enhances EMT in many cancer types, leading to a positive feedback loop [57,58]. Notably, a very recent study has proved that PD-L1 contributes to the transformation of breast cancer cells, with the PD-1/PD-L1 blockade inhibiting the epithelial–mesenchymal transition and improving the chemotherapy response [47].

In our study, a positive correlation was also found between PD-L1 expression levels in TILs and TCs. Indeed, when we consider the aforementioned studies and the results obtained by us, we can hypothesize that higher PD-L1 IHC scores found in TCs may interfere with the cytokine pathway of chemoattraction, an adaptive resistance mechanism of immune escape, suggesting that the PD-1/PD-L1/PD-L2 axis can play a leading role in tumor progression. Although an increasing number of authors focus mainly on the assessment of PD-L1 expression in TCs, the relevance of a comprehensive assessment of PD-L1 expression in TILs is very clear. For example, in HBC, the PD-L1 expression in TILs has been associated with a favorable outcome [28] and with a positive clinical response to anti-PD-L1 immunotherapy [59]. In our study, positive PD-L1 IHC scores in iTILs and sTILs were found in 96% (46/48) and 100% (48/48) of the immunophenotyped samples, respectively. It was also found that, in most cases (30/48, 63%), iTILs and sTILs showed a final PD-L1 IHC score ≥ 6, reflecting at least 6% of marked cells, with intensities ranging from strong to very strong. These results reinforce the idea that cats with rare mammary carcinomas are promising candidates for anti-PD-1 [60] or anti-PD-L1 therapies, as demonstrated in HBC [23,61,62].

Additionally, in the present study, higher PD-L1 IHC scores in iTILs were found to be associated with ulcerated tumors. Notably, it is reported that PD-L1-positive TILs are associated with increased levels of activated TILs [28], reflecting an active anti-tumor immunity that, despite being balanced by the PD-1/PD-L1/PD-L2 pathway, is sufficient to generate an inflamed and immunosuppressive tumor microenvironment, capable of damaging tissues, thus leading to ulcer formation and contributing to tumor progression. Interestingly, this association was only found for intratumoral TILs, which are at the forefront of the fight against tumor growth. Again, our results suggest that the PD-1/PD-L1/PD-L2 pathway may play a crucial role in disease progression of feline mammary carcinoma.

Regarding PD-L2, we found a cytoplasmic and nuclear pattern in TCs, as previously reported [17]. In human oncology, many studies have identified PD-L2 expression in various types of tumors, including, but not limited to, breast carcinoma [17], hepatocellular carcinoma [63], lung carcinoma [64], gastric carcinoma [65] and head and neck squamous cell carcinoma (HNSCC) [66]. In the present study, we found that PD-L2 is expressed by TCs in 79% (38/48) of the samples, and by iTILs and sTILs in 100% of samples. The high prevalence of PD-L2 staining reveals that it probably modulates TME of FMC and progression of the disease [17].

In agreement with our findings, a considerable number of publications have confirmed that PD-L2 is highly expressed in several human tumors [67]. In fact, we found that PD-L2 is more frequently expressed by TCs than PD-L1 (79% vs. 46%), raising an important question: does PD-L2 have a more relevant role on the immunosuppressive axis than PD-L1 in FMC? Recently, Qiao et al. found that PD-L2 overexpression is dramatically more prevalent than PD-L1 expression in HNSCC, with PD-L2 positivity being observed in 62.7% of patients. Here, the authors hypothesize that PD-L2 could be a potential immunotherapeutic target [66], corroborating our results.

In addition, we also found that 42% (20/48) of the tumors showed PD-L1 and PD-L2 co-expression in TCs and noticed a positive correlation between PD-L1and PD-L2 IHC scores in TCs, corroborating the results recently reported that recognized the existence of a super-enhancer located between the PD-L1 and PD-L2 genes (called PD-L1L2-SE) and which found that PD-L1L2-SE drives the PD-L1 and PD-L2 co-expression by TCs [68]. Therefore, taking these insights into account, we hypothesized that PD-L1 and PD-L2 co-expression in FMC is probably due to the regulatory role of PD-L1L2-SE, as PD-L1 and PD-L2 genes are closely located, as they are in humans, although further studies should be conducted to validate this hypothesis. Additionally, in a study involving 172 head and neck squamous cell carcinoma patients treated with Pembrolizumab (an anti-PD-1 monoclonal antibody), it was found that the overall response rate was twice as high in cancer patients positive for both ligands, compared with patients only PD-L1-positive [69], emphasizing that PD-L2 expression may also stratify cats with mammary carcinoma most likely to benefit from anti-PD-1 immunotherapy.

Moreover, our results reveal that the highest PD-L2 IHC scores in TCs were associated with the absence of skin ulceration. This finding is consistent with previous studies, suggesting that PD-L2 overexpression is responsible for the progression of advanced malignant tumors with high proliferation capacity [67]. This means that tumors with PD-L2 overexpression can more effectively suppress the local immune response, which may result in a less inflamed tumor microenvironment and, consequently, have a lower propensity for ulceration. Furthermore, it has been reported that PD-L2’s binding affinity to PD-1 is two- to six-fold higher than that of PD-L1 [67], suggesting that PD-L2 elicits a higher inhibition of TILs when coupled to the PD-1 receptor, partially justifying the negative correlation obtained for the two ligands. In addition, several reports have shown that PD-L2 overexpressing breast tumors have an enriched epithelial–mesenchymal transition signature, crosslinking immune evasion and EMT [70], whereas in osteosarcoma cells, PD-L2 promotes tumor invasion and metastasis through the RhoA-ROCK-LIMK2 pathway [71], highlighting the importance of this ligand as a potential therapeutic target.

Finally, despite the novelty and importance of the above results, this study has some limitations. Firstly, this is the first study to analyze PD-L2 expression in feline mammary tumor tissue samples. Consequently, carrying out the IHC technique proved to be difficult, as there are no standardized staining and analysis protocols to date. Secondly, for most of the animals, the clinical data were absent, limiting the draw of DFS and OS curves to avoid type 1 or type 2 statistical errors.

## 5. Conclusions

The results of this study show that the PD-1, PD-L1, and PD-L2 proteins are expressed in the analyzed samples, underscoring their importance in FMC, particularly PD-L2. In addition, the expression patterns of each protein in TCs, iTILs, or sTILs may contribute to predicting different clinical outcomes in cats with rare mammary carcinomas.

While the blockade of the PD-1/PD-L1/PD-L2 immunoregulatory axis holds promise as an immunotherapeutic approach for rare FMC subtypes, further investigations are needed, particularly examining the interplay between this pathway and the tumor microenvironment. Evaluations of immune checkpoint inhibitors are also crucial for advancing therapeutic strategies in feline oncology.

## Figures and Tables

**Table 1:** Signalment of cats and clinical and pathological features in rare feline mammary carcinomas included in the study.

Clinicopathological Feature	No of Animals (%) (n = 47) *	Histopathological Feature	No of Samples (%) (n = 48) **
**Age (years)**		**HP classification**	
<8 years old	3 (6%)	Mucinous carcinoma	36 (75%)
8–12 years old	21 (45%)	Adenosquamous carcinoma	8 (17%)
>12 years old	21 (45%)	Carcinosarcoma	4 (8%)
Unknown	2 (4%)	**Histological** **grade**	
**Breed**		I	1 (2%)
European shorthair	33 (70%)	II	10 (21%)
Siamese	2 (4%)	III	37 (77%)
Domestic	2 (4%)	**Tumor necrosis**	
Persian	3 (7%)	Yes	33 (69%)
Norwegian Forest	1 (2%)	No	15 (31%)
Birman	1 (2%)	**Lesion distribution**	
Not determined	5 (11%)	Unifocal	26 (54%)
		Multifocal	22 (46%)
**Clinicopathological** **feature**	**No of samples (%)** (n = 48) **	**Vascular permeation**	
**Tumor size (cm)**		Yes	8 (17%)
<2 cm	9 (19%)	No	40 (83%)
≥2 cm	39 (81%)	**Lymph node status**	
**Tumor ulceration**		Positive	12 (25%)
Yes	10 (21%)	Negative	20 (42%)
No	38 (79%)	Unknown	16 (33%)

* n = 47 corresponds to the 47 female cats included in the study; ** n = 48 corresponds to the 48 feline mammary carcinoma tissue samples; HP—histopathological.

**Table 2:** Results of the statistical analysis between PD-1 IHC scores in different cell types and clinicopathological features, using the Mann–Whitney and Kruskal–Wallis test.

Clinicopathological Features	PD-1 IHC Score					
	TCs Md (IQR)	*p* Value	iTILs Md (IQR)	*p* Value	sTILs Md (IQR)	*p* Value
Age (years) **						
<8 years old	0 (nd)	0.23	3 (nd)	0.34	3 (nd)	0.74
8–12 years old	0 (0)		6 (3)		6 (6)	
>12 years old	0 (0)		4 (4)		6 (3)	
Breed **						
European shorthair	0 (0)	0.17	4 (4)	0.96	4 (4)	0.57
Siamese	0 (0)		6 (nd)		6 (nd)	
Domestic	0 (0)		4 (nd)		6 (nd)	
Persian	0 (nd)		3 (nd)		9 (nd)	
Norwegian Forest	4 (0)		4 (0)		4 (0)	
Birman	0 (0)		6 (0)		9 (0)	
Not determined	0 (2)		6 (2)		6 (4)	
**Tumor size (cm)**						
<2 cm	0 (0)	0.98	4 (5)	0.72	6 (5)	0.82
≥2 cm	0 (0)		4 (4)		6 (5)	
**Tumor ulceration**						
Yes	0 (0)	0.83	6 (4)	0.35	6 (2)	0.77
No	0 (0)		4 (4)		5 (7)	
HP classification **						
Mucinous carcinoma	0 (9)	0.72	4 (4)	0.65	6 (4)	0.99
Adenosquamous carcinoma	0 (0)		5 (4)		6 (6)	
Carcinosarcoma	0 (0)		7 (7)		5 (6)	
Tumor histological grade **						
I	0 (0)	**0.012**	4 (0)	0.70	4 (0)	0.85
II	0 (4)		4 (5)		5 (5)	
III	0 (0)		6 (4)		6 (5)	
**Tumor necrosis**						
Yes	0 (0)	**0.033**	4 (4)	0.21	4 (4)	0.06
No	0 (4)		6 (2)		6 (5)	
**Lesion distribution**						
Unifocal	0 (0)	0.14	4 (4)	0.61	6 (6)	0.45
Multifocal	0 (0)		5 (3)		4 (4)	
**Vascular permeation**						
Yes	0 (0)	0.95	4 (4)	0.56	6 (4)	0.49
No	0 (0)		5 (4)		6 (5)	
**Lymph node status**						
Yes	0 (0)	0.92	6 (4)	0.25	6 (4)	0.14
No	0 (0)		4 (4)		4 (4)	

** Characteristics that were evaluated using the Kruskal–Wallis test. *p* values in bold are statistically significant (*p* < 0.05). (nd)—IQR undetermined. iTILs—Intratumoral tumor-infiltrating lymphocytes; sTILs—stromal TILs; TCs —tumor cells; Md—median; IQR—interquartile range; HP—histopathological.

**Table 3:** Results of the statistical analysis between PD-L1 and PD-L2 IHC scores in different cell types and clinicopathological characteristics, using the Mann–Whitney and Kruskal–Wallis tests.

Clinicopathological Features	PD-L1 IHC Score						PD-L2 IHC Score					
	TCs Md (IQR)	*p* Value	iTILs Md (IQR)	*p* Value	sTILs Md (IQR)	*p* Value	TCs Md (IQR)	*p* Value	iTILs Md (IQR)	*p* Value	sTILs Md (IQR)	*p* Value
Age (years) **												
<8 years old	6 (nd)	0.59	6 (nd)	0.56	6 (nd)	0.51	6 (nd)	0.08	6 (nd)	0.71	6 (nd)	0.27
8–12 years old	2 (5)		6 (5)		6 (5)		4 (3)		6 (4)		6 (3)	
>12 years old	0 (6)		6 (2)		6 (5)		2 (4)		6 (3)		8 (4)	
Breed **												
European shorthair	0 (4)	0.12	6 (5)	0.75	6 (2)	0.29	4 (4)	0.98	6 (5)	0.62	6 (5)	0.67
Siamese	6 (nd)		6 (nd)		9 (nd)		6 (nd)		6 (0)		8 (nd)	
Domestic	2 (nd)		4 (nd)		4 (nd)		3 (nd)		5 (nd)		6 (0)	
Persian	2 (nd)		4 (nd)		6 (nd)		4 (nd)		6 (nd)		6 (nd)	
Norwegian Forest	3 (0)		6 (0)		6 (0)		4 (0)		6 (0)		6 (0)	
Birman	6 (0)		6 (0)		12 (0)		4 (0)		3 (0)		4 (0)	
Not determined	0 (3)		6 (5)		6 (7)		2 (5)		6 (5)		8 (3)	
**Tumor size (cm)**												
<2 cm	0 (5)	0.87	4 (3)	0.07	4 (7)	0.96	4 (4)	0.14	9 (4)	0.36	6 (4)	0.74
≥2 cm	0 (6)		6 (5)		6 (4)		4 (3)		6 (5)		6 (5)	
**Tumor ulceration**												
Yes	1 (6)	0.61	9 (3)	**0.009**	6 (4)	0.18	2 (4)	**0.048**	6 (4)	0.83	6 (2)	0.83
No	0 (5)		6 (2)		6 (3)		4 (4)		6 (5)		6 (5)	
HP classification **												
Mucinous carcinoma	3 (6)	0.16	6 (2)	0.30	6 (5)	0.56	4 (4)	0.43	6 (5)	0.63	6 (5)	0.58
Adenosquamous carcinoma	0 (5)		6 (5)		6 (2)		2 (4)		6 (3)		7 (3)	
Carcinosarcoma	0 (0)		8 (5)		6 (2)		4 (2)		8 (8)		5 (4)	
Tumor histological grade **												
I	0 (0)	0.58	4 (0)	0.21	4 (0)	0.45	2 (0)	0.13	2 (0)	0.18	4 (0)	0.44
II	2 (6)		4 (2)		5 (3)		6 (5)		6 (5)		6 (4)	
III	0 (5)		6 (5)		6 (5)		4 (3)		6 (3)		6 (4)	
**Tumor necrosis**												
Yes	0 (6)	0.57	6 (5)	0.18	6 (5)	0.76	0 (6)	0.15	6 (4)	0.16	6 (5)	0.61
No	0 (4)		4 (6)		6 (5)		0 (4)		9 (3)		6 (3)	
**Lesion distribution**												
Unifocal	0 (6)	0.57	6 (5)	0.82	6 (2)	0.06	4 (3)	0.48	6 (4)	0.62	6 (5)	0.64
Multifocal	0 (4)		6 (5)		4 (5)		3 (5)		6 (5)		6 (4)	
**Vascular permeation**												
Yes	0 (3)	0.42	8 (5)	0.14	6 (5)	0.33	1 (6)	0.23	6 (0)	0.88	9 (3)	0.18
No	0 (6)		6 (2)		6 (4)		4 (2)		6 (5)		6 (5)	
**Lymph node status**												
Yes	1 (6)	0.39	6 (5)	0.35	6 (5)	0.69	1 (4)	0.24	6 (2)	0.21	7 (3)	0.37
No	0 (4)		6 (2)		6 (2)		4 (2)		6 (4)		6 (5)	

** Characteristics that were evaluated using the Kruskal-Wallis test. *p* values in bold are statistically significant (*p* < 0.05). (nd)—IQR undetermined. iTILs—Intratumoral tumor-infiltrating lymphocytes; sTILs—stromal TILs; TCs —tumor cells; Med—median; AIQ—interquartile range; HP—histopathological.

## Data Availability

The original contributions presented in this study are included in the article. Further inquiries can be directed to the corresponding author.
